# Towards Sustainable Pest Management: Toxicity, Biochemical Effects, and Molecular Docking Analysis of *Ocimum basilicum* (Lamiaceae) Essential Oil on *Agrotis ipsilon *and *Spodoptera littoralis *(Lepidoptera: Noctuidae)

**DOI:** 10.1007/s13744-024-01137-6

**Published:** 2024-03-13

**Authors:** Mona Awad, Nawal Abdulaziz Alfuhaid, Alia Amer, Nancy N. Hassan, Moataz A. M. Moustafa

**Affiliations:** 1 Dept of Economic Entomology and Pesticides, Faculty of Agriculture, Cairo Univ, Giza, Egypt; 2 Dept of Biology, College of Science and Humanities, Prince Sattam Bin Abdulziz Univ, Al-Kharj, Saudi Arabia; 3 Medicinal and Aromatic Plants Dept, Horticulture Research Institute, Agricultural Research Center, Giza, Egypt

**Keywords:** Essential oils, Noctuidae pests, Toxicity, Detoxifying enzymes, Glutathione S-transferase (GST), Molecular docking

## Abstract

Over the last decade, essential oils (EOs) have become potential ingredients for insecticide formulations due to their widespread availability and perceived safety. Therefore, this study aimed to evaluate the toxicity and biochemical efficacy of basil (*Ocimum basilicum*) (Lamiaceae) against two destructive pests Noctuidae, *Agrotis ipsilon* (Hufnagel) and *Spodoptera littoralis* (Boisduval) (Lepidoptera: Noctuidae). In addition, a molecular docking study was performed to gain insight into the binding pattern between glutathione S-transferase (GST) and linalool, the main component of EO. GC–MS analysis of *O. basilicum* EO revealed that linalool is the most abundant compound (29.34%). However, the toxicity tests showed no significant difference between the values of LC_50_ of *O. basilicum* EO to *A. ipsilon* and *S. littoralis*. On the other hand, the sublethal experiments indicated that treating the second instar larvae with LC_15_ or LC_50_ values of *O. basilicum* EO significantly prolonged the larval duration in both insects, compared to the control. Regarding the biochemical effect of *O. basilicum* EO, the treatments significantly impacted the activity of detoxification enzymes. A notable elevation in glutathione S-transferase (GST) activity was recorded in *A. ipsilon* larvae compared with a reduction in *S. littoralis* larvae. The molecular docking analysis revealed that linalool bonded with the amino acid serine (SER 9) of GST, indicating its binding affinity with the enzyme. The obtained results could offer valuable insights into the mode of action of *O. basilicum* and can encourage the adoption of sustainable pest control practices that incorporate essential oils.

## Introduction

Insects are critical in causing global crop losses due to their herbivorous nature and/or being disease vectors (Lucena-Leandro et al. 2022). These arthropods are accountable for decreasing worldwide food production by 20% as well as reducing household food security at the post-harvest level (Sharma et al. 2017). Therefore, adaptive interventions are required, particularly in the context of the impact of Hawkins climate change (IPPC 2021). Conventional pesticides have been used frequently to control agricultural insect pests, and this presents such drawbacks as short time of effectiveness in the field, selection of resistant pest populations, and high toxicity to non-target organisms (Desneux et al. 2007; Khan et al. 2010; Roush and Tabashnik 2012; Lamberth et al. 2013; Gill and Garg 2014; Hawkins et al. 2019). These drawbacks have spurred demand for long-lasting and more eco-friendly alternatives to traditional pesticides.

The development of biologically derived pesticides is a promising approach to discovering novel pesticides or formulation technologies (Abdollahdokht et al. 2022). Considering this, the last decade has seen tremendous efforts to develop environmentally friendly and effective alternatives, with a particular focus on plant extracts (Isman 2020; Palermo et al. 2021; Li et al. 2022; Chatterjee et al. 2023). Botanicals have been recognized as efficient pest control agents, with plant essential oils (EOs) being their most emphasized category (Rathore 2017; Passos et al. 2022). EOs are volatile compounds extracted from species of aromatic plants mainly belonging to the Myrtaceae, Lamiaceae, Lauraceae, and Asteraceae families (Regnault-Roger 1997; Cagá et al. 2022). The extracted essential oils contain aroma-producing compounds such as monoterpenes, phenols, sesquiterpenes, oxides, aldehydes, esters, and ketones (Yong-Lak and Jun-Hyung 2016). The impetus for the use of EOs is associated with the constitutive advantages of their properties, low toxicity to mammals, and little persistence in the environment. Therefore, EOs have been proposed for organic and integrated pest management programs (Campolo et al. 2017; Pavela et al. 2020). The Lamiacea family has been validated for its insecticidal potential (Prasannakumar et al. 2023). The *Ocimum basilicum* is widely distributed in Egypt (Kandil et al. 2009) and is known for their significant medicinal values (Vasudevan et al. 1999). Several studies reported that chemical components of *O. basilicum* showed insecticidal properties against insect pests such as *Spodoptera litura *(Fabricius) and *Rhyzopertha dominica* (Fabricius) (Hummelbrunner and Isman 2001; Ebadollahi et al. 2022).

The family Noctuidae has received great attention because it contains serious pests on a wide range of agricultural plants (Zuo et al. 2022a and b; Henaish 2023). They are believed to be the most destructive pests of vegetables, destroying gardens, orchards, and crops every year (Capinera 2008; Zahiri et al. 2012). One of the most harmful and destructive Noctuid pests is *Spodoptera littoralis *(Boisduval) the Egyptian cotton leafworm. It exists throughout the year and infests about 90 plant species belonging to 40 plant families including cotton, the main economic crop in Egypt (Shaurub et al. 2020). Recently, populations of *S. littoralis* with high levels of resistance to several groups of insecticides were selected and this pest is ranked among the top 30 highly resistant species worldwide, as listed by the Arthropod Pesticide Resistance Database (http://www.pesticideresistance.org, accessed on 18 May 2021). On the other hand, black cutworm *Agrotis ipsilon* (Hufnagel) is a major subterranean pest. The larvae hide in the soil and feed on the stems of seedlings, resulting in damaged growth and plant death (Xiang et al. 2010). It is difficult to manage this pest using traditional insecticides because of its resistance and its nocturnal activity (Li et al. 2007).

Conversely, insects develop multiple strategies to overcome the potential toxicity of these xenobiotics (Després et al. 2007; Hu et al. 2019; El-Sayed et al. 2023). The insect detoxification enzyme system includes three phases: biotransformation, metabolism, and secretion of insecticides before reaching the target sites and producing their toxic effects (Li et al. 2007; Xu et al. 2020; You et al. 2023). Phase I detoxifying enzymes include cytochrome P450 monooxygenases, esterases, and flavin monooxygenases, which catalyze the responsible oxidation, reduction, and hydrolytic reactions, as well as incorporate polar groups to enhance the water solubility of toxic molecules (Liao et al. 2016). Phase II enzymes, including glutathione S‐transferases (GSTs), UDP‐glucuronosyltransferases (UGTs), and sulfotransferase, conjugate the molecules to improve the water solubility of phase I products (Aioub et al. 2023). Phase III transporters, such as adenosine triphosphate–binding cassette (ABC), export the conjugated toxins from the cell (Tijet et al. 2001; Liu et al. 2015).

In general, the insecticidal activity of EOs has been frequently assessed against insect pest species (Benelli et al. 2018). However, as rare studies explored the biochemical targets and intermediate changes, the mode of action of EO is still in need of deep understanding (Hashem et al. 2020).

To mitigate some of the drawbacks associated with the use of EOs in pest management programs, the current study aimed to evaluate the toxicity and biochemical efficacy of basil (*Ocimum basilicum)* against *A. ipsilon* and *S. littoralis*. In addition, to gain insights into the binding pattern between linalool, the major EO constituent, and GST, we conducted a molecular docking study.

## Materials and Methods

### Insect Colony

*Agrotis ipsilon* and *S. littoralis* cultures were provided by the Entomology Department, Faculty of Agriculture, Cairo University, Giza, Egypt (30.0131°N, 31.2089°E). Both insects were raised in sterile plastic containers (17 × 25 × 8 cm) under suitable conditions (8 h darkness: 16 h light at 25 °C and 60% relative humidity) (Moustafa et al. 2021a, 2023a; Awad et al. 2022). *Agrotis ipsilon* larvae were raised separately (Moustafa et al. 2021a) in small plastic cups and fed on fresh castor leaves until pupation. The pupae were maintained in glass jars with paper tissues until adult emergence. Bioassays were carried out on the 2nd instar larvae under suitable laboratory conditions (Moustafa et al. 2021a, 2022).

### Basil, Ocimum basilicum, Oil

Basil oil samples were obtained from the Medicinal and Aromatic Plants Research Department, El-Qanater El-Khairiya, Qalubeia Governorate, Egypt (30°19′N, 31°13′E, 16.9 m above sea level). Extraction was carried out according to Moustafa et al. (2023a) and the obtained EO was dried and stored in sealed Eppendorf tubes until use.

### Gas Chromatography–Mass Spectrometry (GC–MS) Analysis

Identification of the chemical composition of *O. basilicum* EO was done as described by Moustafa et al. (2021a). Shimadzu single quadrupole gas chromatograph-mass spectrometer (GC–MS-QP) 2015 plus (Kyoto, Japan) was used via 0.5 µL injections of the EO on a Hewlett Packard chromatograph model 597 equipped with a flame ionization detector (FID) and a 50-cm HP capillary column. For identification, the retention time (RT) of each obtained peak was compared with the data in the WILEY/NIST and Tutor Libraries (Beckley et al. 2014; Abd El-Kareem et al. 2016).

### Toxicity

The lethal and sublethal concentrations of *O. basilicum* EO were estimated. Second instar larvae of *S. littoralis* and *A. ipsilon* were treated with five concentrations, viz 8000, 4000, 2000, 1000, and 500 mg/L). For each concentration, five replicates were used (10 larvae/replicate). Castor bean leaves were dipped in each concentration for 20 s then left to air-dry (Hamada et al. 2018), while other leaves were dipped in water for control group. The larvae fed on the treated leaves for 24 h and the survivors were kept in a clean jar supplied with fresh untreated leaves. The larval mortality was recorded daily (Moustafa et al. 2021a) after the correction with the natural death rate in the experiment using Abbott’s formula (Abbott 1925). The toxicity experiment was repeated twice.

### Lethal and Sublethal Effects

The sublethal effect of basil EO on the development of both insects was evaluated using the estimated LC_15_ and LC_50_. Three replicates, each containing fifty larvae, were used for each concentration. The surviving larvae were kept in a tiny, dry cups containing fresh, untreated castor bean leaves (Moustafa et al. 2021b and 2023b) and the developmental changes were recorded daily. The developmental changes were evaluated based on the following variables: larval and pupal duration (days), pupation percentage, pupal weight (g), sex ratio, and adult emergence rate.

To calculate percentages of fecundity and hatchability, three replicates were used (five females and seven males/replicate) (Moustafa et al. 2016 and 2023b).

### Biochemical Assay

#### Sample Preparation

The 2nd instar larvae were treated with the LC_15_ and LC_50_ estimated values of *O. basilicum* EO. The detoxifying enzymes’ activity was assessed after 24, 48, 72, and 96 h of treatment using 50 mg of the fresh body weight of the surviving larvae (Moustafa et al. 2023a). Five replicates were used for each concentration. The larvae were homogenized in 0.1 M phosphate buffer with pH 7.0 for carboxylesterase (CarE), pH 7.4 for cytochrome P450 (P450), and pH 6.5 for glutathione S-transferase (GST). The supernatants from the homogenates were transferred into clean sterile tubes (each of 1.5 mL) after a 15-min centrifugation at 7000 rpm.

#### Carboxylesterase Assay

CarE activity (α- and β-esterase) was assessed according to the methods outlined by van Asperen (1962) and Moustafa et al. (2023a). Alpha- or beta-naphthyl acetate (30 mM) was added to the homogenate sample and the mixture was left for 15 min at 25 °C. Fast Blue b (2%) and sodium dodecyl sulfate (5%) were added to stop the reaction. For α- and β- esterase, the optical density was measured at 550 and 600 nm, respectively, using a Jenway-7205UV/Vis Spectrophotometer.

#### Cytochrome P-450 Monooxygenase Assay

As described by Hansen and Hodgson (1971) and Moustafa et al. (2023a), *P*-nitro anisole (PN) was used for measuring cytochrome P-450 activity. A mixture of 100 µL of 2 mM p-nitro anisole and 90 µL of homogenate sample was incubated at 27 °C for 2 min then 10 µL of 9.6 mM NADPH was added. The optical density was determined at 405 nm using a microplate reader (Clindiag-MR-96, ISO09001:2008, Belgium).

#### Glutathione S-Transferase Assay

GST activity was determined in accordance with Habig et al. (1974) and Moustafa et al. (2023a) using 1-chloro-2,4-dinitrobenzene (CDNB). The sample solution consisted of the sample homogenate, 30 mM CDNB, and 50 mM GSH. The GST activity was measured at 340 nm for 5 min at 1-min intervals using a Jenway-7205 UV/V spectrophotometer,

#### Protein Determination

Coomassie brilliant blue assay was used to calculate the protein concentration according to Bradford (1976).

### Molecular Docking Analysis

The interaction and binding between linalool (the most abundant constituent of basil EO) and GST were examined utilizing the molecular docking tests using the MOE 2015 software. The structure of the compounds was created from the output of the Gaussian 09 software in the PDB file format. GST crystal structures (PDB ID: 1PN9) were downloaded from the protein data bank (http://www.rcsb.org.pdb). The most potent complexes, the ligands, were built into 3D structures using Chem Draws 18.0 and saved as MDL molfiles. The compound that had the lowest binding affinity received the highest rating.

### Data Analysis

SPSS (V.22) was used to enter, code, and analyze the data. The data were examined for meeting the criteria for parametric testing. The Shapiro–Wilk and Kolmogorov–Smirnov tests were used to determine the normality of the continuous variables. The probability and percentile data were standardized using the Arcsine Square Root transformation. The data were presented as (mean ± SD). ANOVA was performed for both the control and treatments and the Tukey pairwise post hoc analysis was carried out. *P*-value was considered significant at < 0.05. Chi-square (*χ*^2^) method was used (MiniTab V. 14) to record the observed and expected frequencies of the toxicity. The analysis became available using SigmaPlot (V.12.0) while R studio (V.2022.02.4.) was used for data visualization.

## Results

### Chemical Composition of Ocimum basilicum Essential Oil

**Fig. 1:**
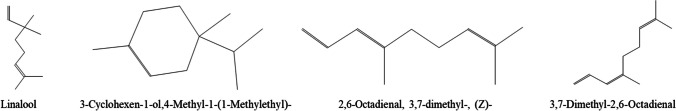
Chemical structure of the main bioactive compounds of basil EO, *O. basilicum*

The chemical compounds of basil EO were identified using GC–MS as shown in Table 1 and Fig. 1. The main bioactive compounds included linalool (29.34%), 3,7-dimethyl-2,6-octadienal (13.16%), 2,6-octadienal, 3,7-dimethyl-, (Z)- (8.82%), and 3-cyclohexen-1-ol,4-methyl-1-(1-methylethyl)- (7.20%).

**Table 1:** The chemical compounds identified in the essential oil from basil, *Ocimum. basilicum*

RT	Area %	Compound name	Match factor (MF)
2.02	0.49	2-Ethyl-oxetane	885
3.54	0.30	Bicyclo[3.1.0]hex-2-ene,4-methyl-1-(1-methylethyl)-	925
3.67	0.97	(1R)-2,6,6-Trimethylbicyclo[3.1.1]hept-2-ene	920
4.41	0.27	Bicyclo[3.1.1]heptane,6,6-dimethyl-2-methylene-	929
4.48	0.47	5-Hepten-2-one, 6-methyl-	873
5.32	1.64	o-Cymene	930
5.46	3.82	Eucalyptol	939
6.34	1.11	Cyclohexanol,1-methyl-4-(1-methylethenyl)-, cis-	846
6.70	0.77	2-Furanmethanol,5-ethenylterahydro-à, à,5-trimethyl-,trans	917
7.08	29.34	Linalool	927
8.16	1.01	Bicyclo [2.2.1]heptan-2-one,1,7,7-trimethyl-, (1S)-	905
9.05	7.20	3-Cyclohexen-1-ol,4-methyl-1-(1-methylethyl)-	936
9.49	1.39	Benzene,1-methoxy-4-(1-propenyl)-, (Z)-	896
9.75	0.37	2-Decenal, (E)-	828
10.20	5.51	2,6-Octadien-1-ol, 3,7-dimethyl-, (Z)-	929
10.50	8.82	2,6-Octadienal, 3,7-dimethyl-, (Z)-	942
10.87	3.12	Geraniol	936
11.28	13.16	3,7-Dimethyl-2,6-octadienal	943
11.46	0.25	2,6-Octadien-1-ol, 3,7-dimethyl-,formate, (Z)-	903
11.64	0.32	Bicyclo[2.2.1]heptan-2-ol,1,3,3-trimethyl-, acetate,endo-	900
12.02	0.31	2,6-Octadien-1-ol, 3,7-dimethyl-,formate, (Z)-	895
13.48	0.87	Phenol,2-methoxy-4-(2-propenyl)-	932
13.56	1.18	2,6-Octadien-1-ol, 3,7-dimethyl-,acetate, (Z)-	871
13.98	0.27	Tricyclo[4.4.0.0(2,7)]dec-3-ene,	926
14.06	0.48	2,6-Octadien-1-ol, 3,7-dimethyl-,acetate	918
14.19	0.32	(-)-á-Bourbonene	757
14.34	0.49	Cyclohexane,1-ethenyl-1-methyl-2,4-bis(1-methylethenyl)-, [1S-(1à,2á,4á)]-	919
15.06	1.98	Caryophyllene	942
15.40	3.81	Bicyclo[3.1.1]hept-2-ene,2,6-dimethyl-6-(4-methyl-3pentenyl)-	954
15.93	0.77	Humulene	933
16.63	0.32	1,6,10-Dodecatriene,7,11-dimethyl-3-methylene-,(E)-	945
16.93	0.40	1,4-Methanoazulen-9-ol,decahydro-1,5,5,8a-tetramethyl-,[1R-(1à,3aá,4à,8aá,9S*)]-	784
17.06	0.31	Azulene,1,2,3,5,6,7,8,8a-octahydro-1,4-dimethyl-7-(1-methylethenyl)-,[1S-(1à,7à,8aá)]-	895
17.21	0.30	2,6,10-Dodecatrien-1-OL,3,7,11-trimethyl-	868
17.34	0.81	Naphthalene,1,2,3,4,4a,5,6,8a-octahydro-7-methyl-4-methylene-1-(1-methylethyl)-,(1à,4aá,8aà)-	946
17.98	2.30	Cyclohexene,4-[(1E)-1,5-dimethyl-1,4-hexadien-1-yl]-1-methyl-	939
18.50	0.21	Nerolidol	897
18.92	1.56	Caryophyllene oxide	917
19.56	0.37	(1R,3E,7E,11R)-1,5,5,8-tetramethyl-12oxabicyclo[9.1.0] dodeca-3,7-dien	895
19.72	0.36	Epicubenol	897
19.85	0.42	10-Epi-ç-eudesmol	935
20.34	1.54	.tau.-Cadinol	920
31.32	0.29	1H-Benzocyclohepten-7-ol,2,3,4,4a,5,6,7,8-octahydro-1,1,4a,7-tetramethyl-, cis-	801

### Toxicity of O. basilicum EO to A. ipsilon and S. littoralis Larvae

LC_15_ and LC_50_ values of *O. basilicum* EO to the second instar larvae of both insects are shown in Table 2. The LC_15_ values were 706.29 and 784.93 mg/L while the LC_50_ values were 2748.04 and 2665.70 mg/L to *A. ipsilon* and *S. littoralis*, respectively (Table 2).

**Table 2:** Toxicity of basil (*Ocimum. basilicum*) essential oil to the second instar larvae of *Agrotis ipsilon* and *Spodoptera littoralis*

Compounds	LC_15_ (mg/L) (95% confidence limits)	LC_50_ (mg/L) (95% confidence limits)	Slope ± SE	χ^2^
*A*. *ipsilon*	706.29 (220.94–1222.27)	2748.04 (1742.33–4092.38)	1.75 ± 0.04	2.14
*S*. *littoralis*	784.93 (454.38–1107.66)	2665.70 (2045.53–3529.39)	1.95 ± 0.28	4.47

### Effect of O. basilicum EO on the Development of A. ipsilon and S. littoralis

**Fig. 2:**
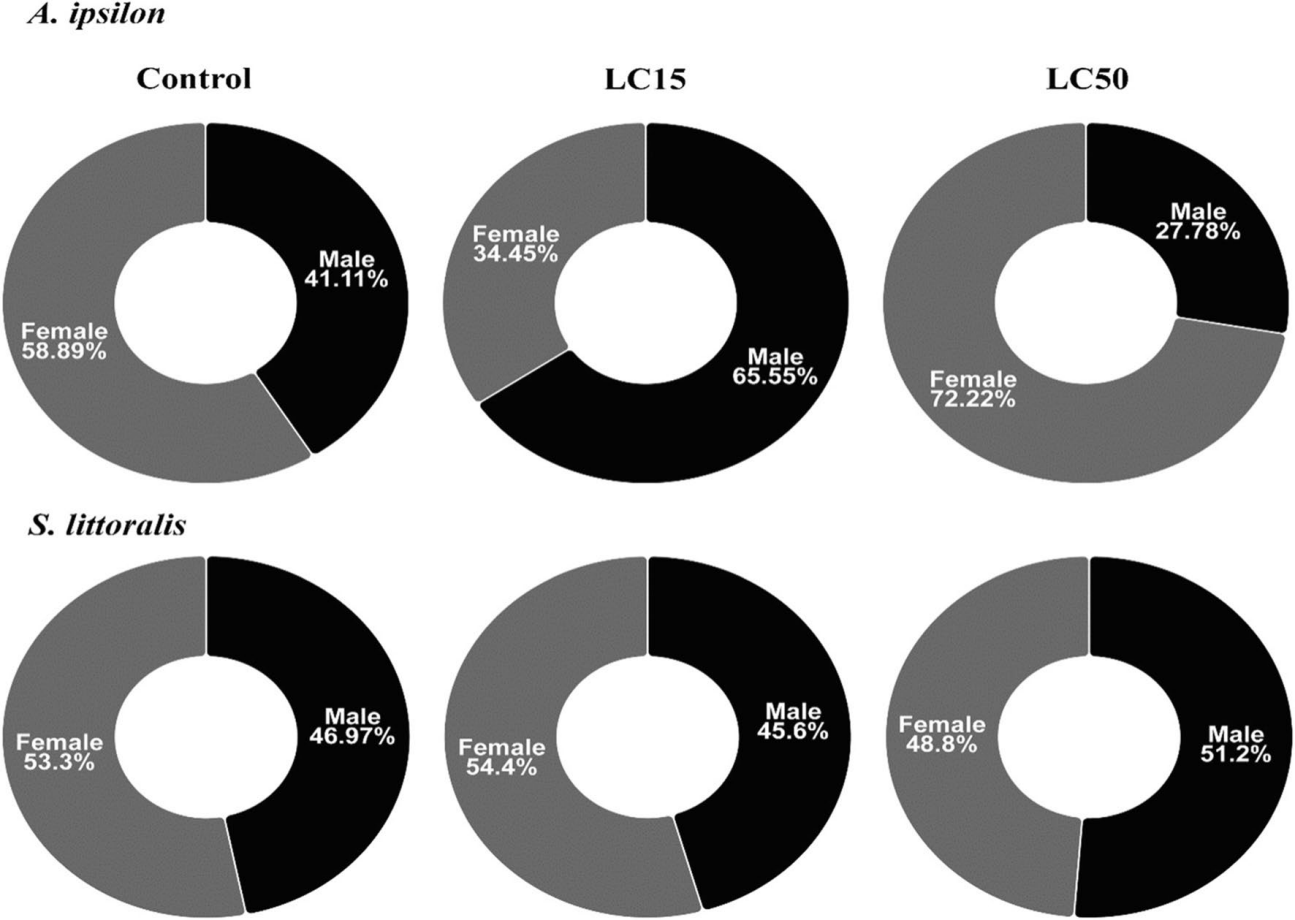
Sex ratio of the emerged adults of *Agrotis ipsilon* and *Spodoptera littoralis* after treating the 2nd instar larvae with LC_15_ and LC_50_ of *Ocimum basilicum* essential oil

As shown in Table 3, when the 2nd instar larvae of both insects were treated with the LC_15_ and LC_50_ of *O. basilicum*, a highly significant elongation in the larval duration (*F* = 25.63; df = 3, 378; *P* < 0.0001) was recorded. Nevertheless, no significant difference (*P* > 0.05) was observed in the pupal duration except for the case of LC_50_ with *A. ipsilon*. In addition, no significant difference was found in pupation (*F* = 0.39; df = 2, 17; *P* = 0.682), emergence (*F* = 0.14; df = 2, 17; *P* = 0.874), or female pupal weight (*F* = 6.12; df = 2,169; *P* = 0.003). Instead, the male pupal weight (*F* = 0.33; df = 2, 173; *P* = 0.721) of *S. littoralis* decreased after treating the larvae with LC_15_ and LC_50_ values. The proportion of emerged females of *A. ipsilon* slightly decreased (by 0.58-fold) after the treatment of the second instar larvae with LC_15_ (LC_15_: χ^2^ = 4.26; *P* = 0.039) while it slightly increased (by 1.22-fold) after the treatment with LC_50_ (χ^2^ = 4.17; *P* = 0.041). As shown in Fig. 2, the same pattern was recorded for *S. littoralis* (LC_15_: χ^2^ = 0.22; *P* = 0.642 and LC_50_: χ^2^ = 0.06; *P* = 0.814).

**Table 3:** Effect of basil (*Ocimum basilicum*) essential oil on the development of *Agrotis ipsilon* and *Spodoptera littoralis* after treating the 2nd instar larvae with LC_15_ and LC_50_ values

Developmental parameters	Mean ± SD					
	*A. ipsilon*			*S. littoralis*		
	Control	LC_15_	LC_50_	Control	LC_15_	LC_50_
Larval duration (days)	19.70^b^ ± 1.3	21.68^a^ ± 1.42	21.96^a^ ± 1.28	16.59^b^ ± 1.71	16.52^b^ ± 1.19	17.65^a^ ± 0.95
Pupal duration (days)	17.51^b^ ± 1.69	18.42^b^ ± 2.06	20.20^a^ ± 1.8	13.15^a^ ± 1.45	13.0^a^ ± 1.34	13.10^a^ ± 1.5
Pupation (%)	100^a^	95.83^a^ ± 2.94	90.60^a^ ± 7.43	95.53^a^ ± 4.15	96.63^a^ ± 2.73	92.20^a^ ± 8.73
Male pupal weight (g)	0.35^a^ ± 0.06	0.33^a^ ± 0.06	0.33^a^ ± 0.06	0.27^a^ ± 0.03	0.25^ab^ ± 0.03	0.24^b^ ± 0.03
Female pupal weight (g)	0.36^a^ ± 0.08	0.38^a^ ± 0.07	0.31^a^ ± 0.06	0.28^a^ ± 0.04	0.26^a^ ± 0.03	0.27^a^ ± 0.04
Emergence (%)	98.61^a^ ± 1.96	98.03^a^ ± 2.77	95.23^a^ ± 6.73	100^a^	100^a^	98.86^a^ ± 1.6

Means that do not share a letter in row are significantly different

### Effect of O. basilicum EO on Detoxifying Enzymes

CarE (*a*-esterase and *β*-esterase), cytochrome P-450, and GST activities were assessed after 24, 48, 72, and 96 h of treating the 2nd instar larvae of *A. ipsilon* and *S. littoralis* with *O. basilicum* EO. As shown in Table 4, the CarE activities increased with all treatments in *A. ipsilon*, while were decreased in *S. littoralis* (Table 5). In contrast, *O. basilicum* EO significantly induced the MFO activity in both insects (Table 4 and 5) after 24, 48, and 72 h from treatments. Interestingly, GST activity significantly increased in *A. ipsilon* (Table 4) and decreased in *S. littoralis* (Table 5).

**Table 4:** The activity of detoxification enzymes (carboxylesterase (α- and β-esterase), cytochrome P-450, and GST) after 24, 48, 72, and 96 h of treating the 2nd instar larvae of *Agrotis ipsilon* with LC_15_ and LC_50_ of *Ocimum basilicum* essential oil

Enzymes	Treatments	Mean ± SD			
		Hours after treatments			
		24 h	48 h	72 h	96 h
α-esterase (µmole/mg of protein)	Control	0.08^b^ ± 0.010	0.12^a^ ± 0.020	0.16^a^ ± 0.03	0.19^ab^ ± 0.01
	LC_15_	0.12^ab^ ± 0.010	0.23^a^ ± 0.029	0.23^a^ ± 0.02	0.25^a^ ± 0.04
	LC_50_	0.15^a^ ± 0.034	0.22^a^ ± 0.056	0.19^a^ ± 0.03	0.14^b^ ± 0.03
β-esterase (µmole/mg of protein)	Control	0.14^a^ ± 0.033	0.12^b^ ± 0.022	0.18^b^ ± 0.002	0.25^ab^ ± 0.009
	LC_15_	0.24^a^ ± 0.063	0.34^a^ ± 0.035	0.33^a^ ± 0.033	0.29^a^ ± 0.028
	LC_50_	0.26^a^ ± 0.013	0.29^a^ ± 0.037	0.27^a^ ± 0.024	0.19^b^ ± 0.028
Cytochrome P-450 (µmole/min /mg of protein)	Control	0.007^c^ ± 0.0004	0.008^a^ ± 0.0003	0.005^b^ ± 0.0005	0.011^a^ ± 0.001
	LC_15_	0.011^b^ ± 0.0003	0.012^a^ ± 0.0021	0.008^a^ ± 0.001	0.017^a^ ± 0.001
	LC_50_	0.016^a^ ± 0.0017	0.011^a^ ± 0.0019	0.007^ab^ ± 0.0002	0.012^a^ ± 0.004
GST (µmol/ml/mg of protein)	Control	20.55^b^ ± 5.046	21.23^c^ ± 6.153	35.78^b^ ± 1.80	47.32^b^ ± 5.391
	LC_15_	63.03^a^ ± 17.725	51.44^b^ ± 8.047	93.08^a^ ± 8.10	96.42^a^ ± 11.88
	LC_50_	90.45^a^ ± 13.787	90.0^a^ ± 6.169	86.74^a^ ± 12.17	100.8^a^ ± 7.172

Means that do not share a letter in column are significantly different

**Table 5:** The activity of detoxification enzymes (carboxylesterase (α- and β-esterase), cytochrome P-450, and GST) after 24, 48, 72, and 96 h of treating the 2nd instar larvae of *Spodoptera littoralis* with LC_15_ and LC_50_ of *Ocimum basilicum* essential oil

Enzymes	Treatments	Mean ± SD			
		Hours after treatments			
		24 h	48 h	72 h	96 h
α-esterase (µmole/mg of protein)	Control	0.30^a^ ± 0.032	0.13^a^ ± 0.001	0.17^a^ ± 0.010	0.22^a^ ± 0.057
	LC_15_	0.07^b^ ± 0.005	0.08^b^ ± 0.003	0.11^b^ ± 0.014	0.23^a^ ± 0.037
	LC_50_	0.06^b^ ± 0.013	0.09^b^ ± 0.007	0.09^b^ ± 0.014	0.23^a^ ± 0.017
β-esterase (µmole/mg of protein)	Control	0.37^a^ ± 0.373	0.35^a^ ± 0.035	0.33^a^ ± 0.023	0.49^a^ ± 0.083
	LC_15_	0.14^a^ ± 0.034	0.21^b^ ± 0.045	0.25^b^ ± 0.011	0.29^b^ ± 0.023
	LC_50_	0.18^a^ ± 0.007	0.18^b^ ± 0.017	0.23^b^ ± 0.027	0.26^b^ ± 0.01
Cytochrome P-450 (µmole/min /mg of protein)	Control	0.010^b^ ± 0.0007	0.006^b^ ± 0.0002	0.003^b^ ± 0.0001	0.007^a^ ± 0.0002
	LC_15_	0.010^b^ ± 0.0002	0.008^ab^ ± 0.0002	0.006^a^ ± 0.001	0.014^a^ ± 0.0027
	LC_50_	0.015^a^ ± 0.0017	0.010^a^ ± 0.002	0.006^a^ ± 0.0009	0.008^a^ ± 0.0031
GST (µmol/ml/mg of protein)	Control	19.48^a^ ± 1.23	11.75^a^ ± 1.575	17.41^a^ ± 1.834	19.57^a^ ± 2.951
	LC_15_	4.28^b^ ± 0.579	2.95^b^ ± 0.449	3.30^b^ ± 0.774	3.71^b^ ± 0.705
	LC_50_	5.17^b^ ± 1.128	4.76^b^ ± 1.084	4.84^b^ ± 0.577	3.22^b^ ± 0.722

Means that do not share a letter in column are significantly different

### Docking Investigation

#### Docking on the receptor of GST (PDB ID: 1PN9)

**Fig. 3:**
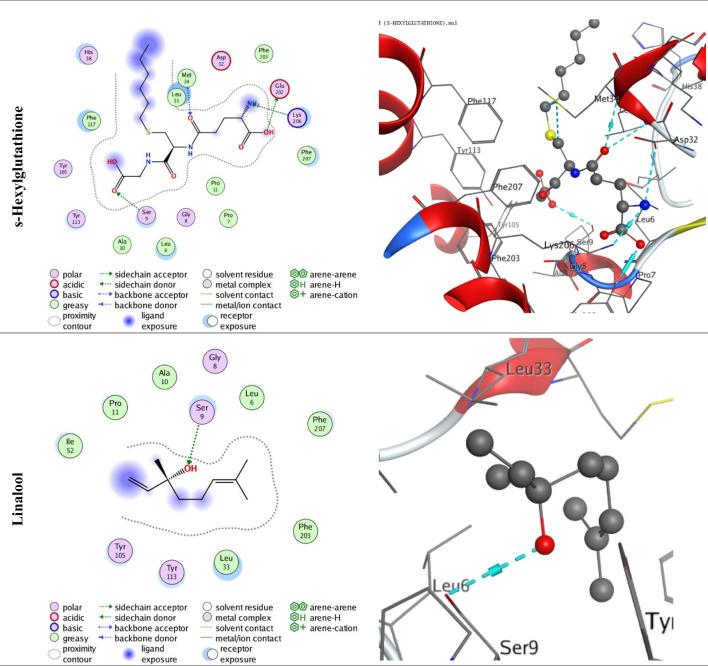
2D and 3D molecular docking simulation of the interactions between the s-Hexylglutathione ligand (GTX) in the enzyme binding pocket and linalool with the active site of the receptor of GST (PDB ID: 1PN9)

The docking procedure (Fig. 3) was initially validated by re-docking with the co-crystallized s-Hexylglutathione ligand (GTX) in the enzyme binding pocket with an energy score (S) of − 4.558 kcal/mol. As shown in Table 6, the docking energy score of the docked compound (linalool) with the enzyme receptor (PDB ID: 1PN9) was − 4.7748 kcal/mol, which is higher than that of the co-crystallized ligand. In addition, linalool bonded with serine (SER 9) residue (Table 6). The overall bonding connections by OH-bonds of the relevant amino acid residue against the docked molecule are depicted in Fig. 3.

**Table 6:** Docking interaction data calculations of co-crystallized s-Hexylglutathione ligand (GTX) in the enzyme binding pocket and linalool with the active site of the receptor of GST (PDB ID: 1PN9)

Compound	Energy score (S) (Kcal/mol)	Affinity bond strength (Kcal/mol)	Affinity bond length (in A^o^ from main residue)	Amino acids	Ligand	Interaction
s-Hexylglutathione	− 4.558	− 4.9	2.61	GLU 202	O 33	H-donor
		− 1.2	3.01	SER 9	O 11	H-acceptor
		− 1.1	2.98	LYS 206	N 29	H-acceptor
		− 1.5	3.18	MET 34	O 36	H-acceptor
Linalool	− 4.7748	− 1.3	2.96	SER 9	O 28	H-acceptor

## Discussion

Insecticide resistance is a critical problem in insect management. Resistance develops through such mechanisms as resistance to penetration, target-site alteration, and enhanced activity of detoxification enzymes (Tangtrakulwanich and Reddy 2014). Accordingly, essential oils have been used as insecticides due to their ability to act on multiple targets. They can enhance the insecticidal effect and are promising as an alternative to traditional insecticides **(**Isman 2020; Duque et al. 2023**)**. In general, the *Ocimum* genus is well known for its insecticidal effect against diverse insect pests (Rodríguez-González et al. 2019). The basic chemical composition of *Ocimum* plants is highly variable and may rely on the genetic properties of the plant and the cultivation conditions (Vieira and Simon 2000). Herein, we analyzed and identified the chemical composition of basil (*O. basilicum*) EO using GC–MS and the analysis revealed that the major constituents were linalool (29.34%), the most abundant compound, 3,7-dimethyl-2,6-octadienal (13.16%), 2,6-octadienal, 3,7-dimethyl-, (Z)- (8.82%), and 3-cyclohexen-1-ol,4-methyl-1-(1-methylethyl)- (7.20%). The results also revealed that this plant may belong to linalool chemotype, which could have a repellent and toxic activities against insects (Rozman et al. 2007; Chaaban et al. 2019).

Regarding the toxicity of *O. basilicum* EO, no significant difference in its LC_50_ values between A. *ipsilon* and *S. littoralis* (about 1.03-fold). Beside toxicity, the sublethal effects on the behavioral and physiological parameters may play a key role in insect pests management (de Araújo et al. 2017). Our experiment showed that the LC_15_ and LC_50_ values of *O. basilicum* EO significantly prolonged the larval duration in both insects, in comparison with the control. Similarly, the pupal durations of *A. ipsilon* were significantly prolonged after administering the 2nd instar larvae with LC_50_ of *O. basilicum* EO. However, no significant differences in the pupal duration of *S. littoralis* were recorded after treating the 2nd instar larvae with LC_15_ and LC_50_, compared to the control.

As to pupation, emergence percentage, and female pupal weight, no significant difference was observed after the treatment of the second instar larvae of both insects with LC_15_ and LC_50_ of *O. basilicum* EO. Nevertheless, the male pupal weight of *S. littoralis* was significantly decreased after treating the larvae with LC_15_ and LC_50_ values. It has been reported that poor nutrition before pupation affected pupa development and prolonged the pupa duration (Aqueel et al. 2015). Earlier studies also confirmed the sublethal effects of chemical or bio-insecticides in a number of lepidopteran pests including *A. ipsilon* (Moustafa et al. 2021a and 2022), *S. littoralis* (Moustafa et al. 2021b and 2023a), *Plutella xylostella* (Linnaeus) (Lepidoptera: Plutellidae) (Wang et al. 2023), *Mamestra brassicae* (Linnaeus) (Lepidoptera: Noctuidae) (Moustafa et al. 2016 and 2023b), and *Tuta absoluta* (Kandil et al. 2020). According to Santos et al. (2017) and Huisamen et al. (2023), the sublethal effect on individuals that is followed by physiological impairment can negatively affect the insect activities and population growth.

Detoxification enzymes are key players in insecticide metabolism in insects (Fouad et al. 2022; You et al. 2022; Aioub et al. 2023; Moustafa et al. 2023c; Prasannakumar et al. 2023). Insect resistance is usually accompanied with enhanced activity of these enzymes (David et al. 2013). In fact, the EOs mode of action needs further understanding. The EOs elicit such distinct neurotoxic symptoms as hyperactivity, agitation, paralysis, and knockdown (Ahmadi et al. 2022). Besides, some studies reported that EOs inhibit detoxifying enzymes (P450s, CarEs, and GSTs) in insects (Tak et al. 2016; Huang et al. 2020). As a target for insecticides, GST is crucial for pesticide detoxification. It converts lipid metabolites of insecticides or combines with toxic molecules via chelation, to protect tissues from oxidative stress (Korkina 2016; Liao et al. 2017). As revealed by our results, GST activities increased significantly by 4.4-, 4.2-, 2.4-, and 2.1-fold after 24, 48, 72, and 96 h of treating *A. ipsilon* larvae with LC_50_ of *O. basilicum* EO. However, a different pattern was recorded for *S. littoralis* larvae, where the LC_50_ of *O. basilicum* EO caused a noticeable inhibition of GST activity 3.8-, 2.5-, 3.6-, and 6.1-fold after 24, 48, 72, and 96 h after treatment, as compared with control. The inhibition of GST can be attributed to the presence of various ingredients in basil EO that act through various modes of action (Liao et al. 2017) while the GST enhanced activity could be an indicator of the adaptation of insects to xenobiotics (Koirala et al. 2022). The cytochrome P450 system protects insects from poisons (Liao et al. 2017). Our study indicated that *O. basilicum* EO significantly induced the cytochrome P450 activity by about 2.3-fold in *A. ipsilon* larvae after 24 h of treatment and by about 1.5-, 1.7-, and twofold in *S. littoralis* larvae after 24, 48, and 72 h of treatment with the LC_50_.

Interestingly, the esterase family of enzymes hydrolyzes ester bonds in insecticides and changes their activities that would result in chemical stress in insects (Gong et al. 2021). Our results showed that the treatment of the 2nd instar larvae with LC_15_ and LC_50_ of *O. basilicum* EO caused a significant increase in *β*-esterase activity in *A. ipsilon* whereas a significant decline was observed in *S. littoralis*, after 48 and 72 h of treatment.

Being more sensitive to essential oils than P450s and CarEs, GST may serve as the primary target of essential oils. Therefore, the decrease in GST activity may be one major cause of insect mortality. In this context, the molecular docking study enables us to specify the most optimal ligands for the GST enzyme. The docking energy score of linalool, the main constituent of basil EO, with the enzyme receptor (PDB ID: 1PN9) was higher than that of the co-crystallized ligand, which confirmed the strong binding between the compound and the receptor. In docking simulations, a lower energy score indicates stronger binding or greater engagement (Shahbaaz et al. 2017). Our current findings are consistent with the experimental findings of in vitro assay. Interaction with the detoxification enzymes is recognized to be the most critical element influencing the biological activity of the compounds against enzymes. For instance, the interaction between citral in *Cymbopogon citratus* EO and cytochrome P-450 enzyme of *S. littoralis* was reported by Moustafa et al. (2023a). Overall, the results could provide better understanding of the mode of action of *O. basilicum* EO at the molecular level, particularly linalool binding affinity with GST receptor.

## Conclusion

In conclusion, *O. basilicum* essential oil demonstrated lethal and sublethal effects against *A. ipsilon* and *S. littoralis*, two Lepidopteran pests severely damaging agricultural production worldwide. In addition, the strong binding between linalool, the main constituent of *O. basilicum* EO, and the GST receptor suggests that GST may be a primary target for *O. basilicum* EO. The obtained results are expected to promote sustainable pest control practices. However, in future investigations, insights into the effects of *O. basilicum* EO under field conditions will be needed to appropriately validate our results.

## Data Availability

The data of the study have been presented in the manuscript.
